# Social Interaction is Unnecessary for Hindgut Microbiome Transmission in Honey Bees: The Effect of Diet and Social Exposure on Tissue-Specific Microbiome Assembly

**DOI:** 10.1007/s00248-022-02025-5

**Published:** 2022-05-02

**Authors:** Kirk E. Anderson, Vincent A. Ricigliano, Duan C. Copeland, Brendon M. Mott, Patrick Maes

**Affiliations:** 1 ARS-USDA Carl Hayden Bee Research Center, 2000 E. Allen Rd., Tucson, AZ 85719 USA; 2 Present Address: ARS-USDA Honey Bee Breeding Genetics and Physiology Research, Baton Rouge, LA 70820 USA; 3 Department of Microbiology, School of Animal & Comparative Biomedical Sciences, University of Arizona, Tucson, AZ 85721 USA; 4 Department of Entomology and Center for Insect Science, University of Arizona, Tucson, AZ 85721 USA

**Keywords:** Mycobiota, Microbiota, Sociality, Honey bee, Gut bacteria, Fungi, Hive environment, Beebread, Pollen

## Abstract

Honey bees are a model for host–microbial interactions with experimental designs evolving towards conventionalized worker bees. Research on gut microbiome transmission and assembly has examined only a fraction of factors associated with the colony and hive environment. Here, we studied the effects of diet and social isolation on tissue-specific bacterial and fungal colonization of the midgut and two key hindgut regions. We found that both treatment factors significantly influenced early hindgut colonization explaining similar proportions of microbiome variation. In agreement with previous work, social interaction with older workers was unnecessary for core hindgut bacterial transmission. Exposure to natural eclosion and fresh stored pollen resulted in gut bacterial communities that were taxonomically and structurally equivalent to those produced in the natural colony setting. Stressed diets of no pollen or autoclaved pollen in social isolation resulted in decreased fungal abundance and bacterial diversity, and atypical microbiome structure and tissue-specific variation of functionally important core bacteria. Without exposure to the active hive environment, the abundance and strain diversity of keystone ileum species *Gilliamella apicola* was markedly reduced. These changes were associated with significantly larger ileum microbiotas suggesting that extended exposure to the active hive environment plays an antibiotic role in hindgut microbiome establishment. We conclude that core hindgut microbiome transmission is facultative horizontal with 5 of 6 core hindgut species readily acquired from the built hive structure and natural diet. Our findings contribute novel insights into factors influencing assembly and maintenance of honey bee gut microbiota and facilitate future experimental designs.

**Supplementary Information:**

The online version contains supplementary material available at 10.1007/s00248-022-02025-5.

## Introduction

A variety of symbiotic microbial associations have developed in the guts of insects and other animals. Social insects in particular present unique opportunities to investigate host–microbiome interactions due to variation in individual behavior, phenotype, diet, and lifespan occurring within the same genetic unit [1–3]. Given the long co-evolutionary relationship between honey bees and their microbiota, the small number of interacting taxa, and the predictability of hindgut microbiota structure, this highly social insect group has become a model for microbiome research [4–6]. However, the social and “built structure” context of honey bee life history [7] is problematic for many experiments making it difficult to gauge individual vs. group effects. Removing the honey bee individual from the colony and hive environment is necessary to test many hypotheses, but such manipulations alter a variety of biotic and abiotic influences on individual physiology, behavior and hindgut microbiota colonization [8, 9].

Honey bee (*Apis mellifera*) colonies are adaptively organized groups of individuals that collect and process floral-derived nutrition via age-based division of labor [10]. Younger adults consume a diet of stored pollen and convert this nutrient source to a fat and protein rich jelly used to feed developing larvae and the queen [11, 12]. Thus, young bees are called nurses. Foraging bees are older, collect pollen, nectar and tree resin, and have significantly lower levels of internal nutrient stores [13]. Spanning this variation in worker task and physiology, an elaborate network of high-frequency trophallactic interactions (mouth to mouth transfer of jelly and sugar rich liquids) intimately connects the nutritional state of the colony and also facilitates microbial transmission [14]. Colony substances produced or collected by honey bees exhibit varying degrees of antimicrobial and antioxidant activities including salivary and hypopharygeal gland secretions, royal jelly, nectar, honey, and stored pollen [12, 15–19]. The core hindgut bacteria are detected consistently throughout all of these niches [20–23]. Although coprophagy has been suggested as a transmission mechanism for core gut bacteria, honey bees do not perform this behavior [8, 9]. However, newly emerged bees immediately sample the hive environment with their mouthparts, an activity that includes cleaning the cells of recently emerged adults, and autogrooming [9, 24–26]. Pollen consumption occurs from 2–10 days of age concurrent with nursing behavior and exposure to much of the social and colony environment [26, 27].

Considering the honey bee gut microbiome from the colony perspective, the interactions among and between individuals and the colony environment are comprised of numerous biotic and abiotic factors influencing one another to varying degrees. This arrangement of social interactions and built structure is considered a “factory in a fortress” [28]. Although this early model largely ignored microbes and disease, the evolution of honey bee sociality was undoubtedly subject to constant microbial challenge at multiple levels including host control of the colony environment [15, 29, 30]. The honey bee harbors a highly predictable microbiota in the hindgut, and colony/hive environment [17, 22, 31, 32]. For the purposes of this research, we define the active colony microbiota as microbes typically found on the mouthparts of workers and queens and throughout the interactive network of food distribution, including the social stomach (crop), the hypopharygeal glands, stored honey, stored pollen (beebread), and larval environments. The core hindgut bacteria are prevalent throughout the colony environment but differ in prevalence by niche [22, 23, 33, 34]. This foundation aids in distinguishing disease transmission from hindgut microbiome transmission and microbes vectored from the pollination environment.

The hindgut of the adult worker honey bee harbors a core hindgut microbial community of five omnipresent bacterial groups totaling approximately 10^8^ -10^9^ bacterial cells [31, 35]. The hindgut microbiome is demarcated by the pylorus, where metabolic waste materials are excreted by the Malpighian tubules into the hindgut environment. Bacterial membership and function differs by hindgut region. The pylorus of healthy bees is often colonized by a sixth bacterium, *Frischella perrara,* causing an immune cascade and melanized scab [36, 37]. *Snodgrassella alvi* interfaces with host epithelium at the ileum, and occurs in a biofilm with *Gilliamella apicola,* and *Lactobacillus* firm 5 [4, 38, 39]. The rectum comprises the largest bacterial community and is dominated primarily by four species of *Lactobacillus* firm 5, and lesser amounts of *Lactobacillus* firm 4 and *Bifidobacterium*. Less understood, and sporadically detected in the worker hindgut are *Lactobacillus kunkeei* and *Bombella apis* (a synonym of *Parasaccharibacter apium*), bacteria found with greater abundance/prevalence in queen guts and/or the active colony environment [17, 23, 40, 41].

In worker bees, the establishment of a typical hindgut microbiota happens in the first few days of adult life, leads to increased weight gain, reduced susceptibility to pathogens, and priming of the host immune system [4, 42, 43]. However, atypical or altered bacterial establishment in early adult life leads to reduced weight, impaired development, disease progression and early mortality [21, 44]. Studies investigating adult hindgut colonization have produced variable results, and the contribution of various factors to hindgut microbiota establishment remains unclear [8, 9, 35, 45, 46]. Larvae shed their gut lining prior to pupation, and are recolonized as adults. Although studies are limited, few microbes are thought to survive this transition [47]. One primary difficulty for experimental design is that adult hindgut assembly happens concurrently with the consumption of stored pollen by newly emerged worker bees. Following adult emergence, honey bees experience multiple social cues, pollen consumption, and exposure to a variety natural colony niches that might affect microbiome establishment [17, 26].

Most recently, the honey bee hindgut microbiota has become the subject of significant research interest due to its role in disease susceptibility [32, 43], a major role played by gut bacteria in most host species. Here we explore hindgut microbiome assembly (transmission and establishment) of the adult worker bee considering social and non-social environments, fungal colonization and diet. We allowed natural eclosion from the natal frame, and assessed microbiome colonization of nine-day-old worker bees for specific gut regions (midgut, ileum and rectum). We used bacterial 16S rRNA gene sequencing, fungal specific 18S sequencing and qPCR to quantify treatment effects on microbiota colonization and character. We discuss the evolutionary implications of our findings and provide perspective for the interpretation and refinement of honey bee microbiome experiments.

## Methods

### Experimental Design

**Fig. 1:**
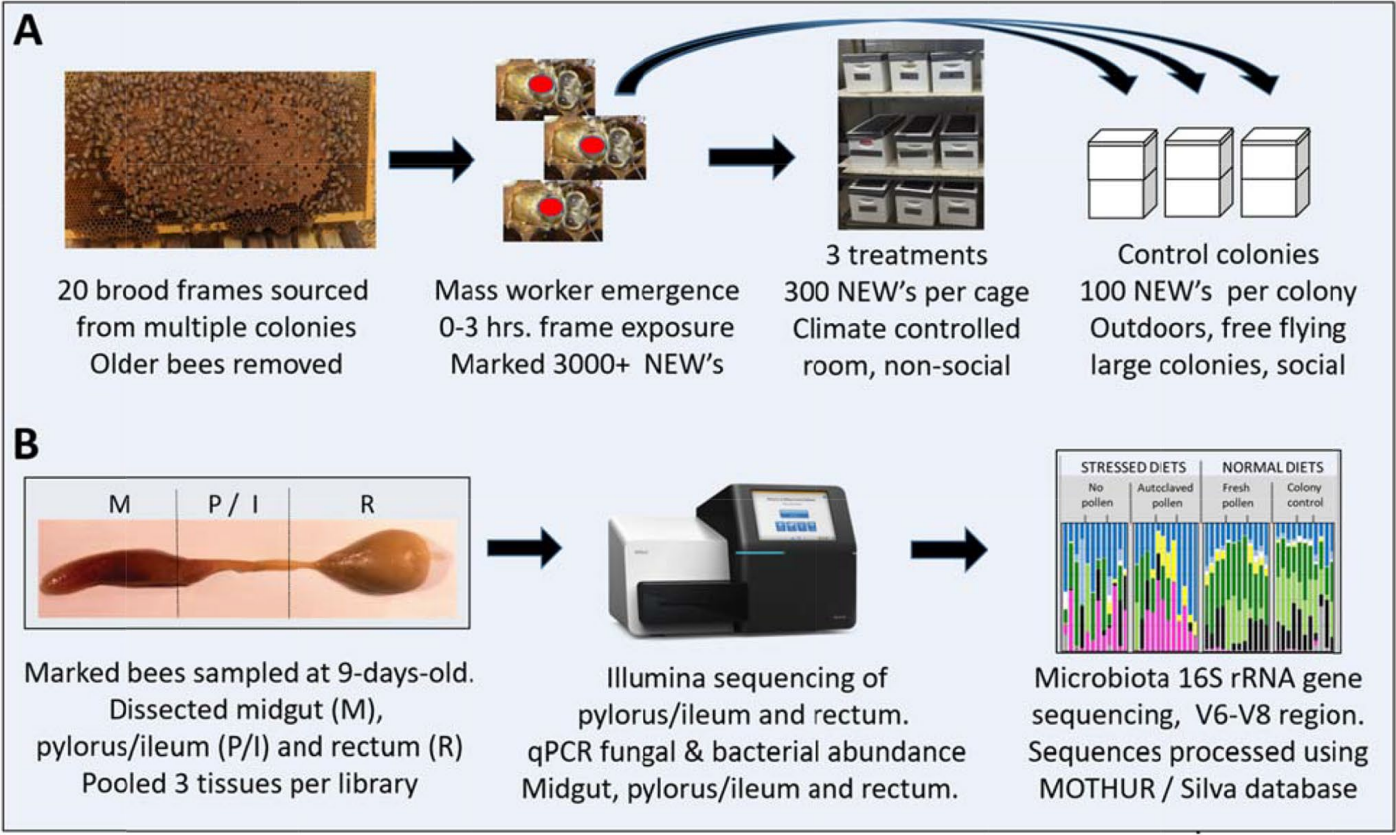
Summary of experimental approach. (**A**) Highlights the marked bee assay and treatment/control conditions. Newly emerged bees (NEW’s) were collected from a climate controlled room and marked with a dot of paint on their thorax. The three treatments were fresh pollen, autoclaved pollen, and no pollen, all applied in social isolation in a climate controlled room. All treatment cages were provided autoclaved sucrose syrup and water ad libitum. Control colonies contained over 10,000 established (older) workers and were exposed to the pollination environment. (**B**) Illustration of tissue dissection and molecular analyses. The pylorus and ileum (P/I) were dissected, sequenced and analyzed as a unit

We gathered emerging brood frames from 16 actively growing colonies in July 2016, at the Carl Hayden Bee Research Center in Tucson AZ. Twenty capped brood frames containing a high proportion of dark-eyed pupae were placed into screened collection boxes under controlled climate conditions [48]. We allowed natural eclosion, defined as newly emerged workers (NEWs) chewing out of their natal cocoon and capped cell, and performing early life behaviors on their natal wax frame including larval cell cleaning [9, 26]. All NEWs were exposed to naturally occurring colony components beebread, honey, wax frame and empty development cells for 0–3 h (Fig. 1).

We tested the hypothesis that establishment of the hindgut microbiota in NEWs relies on contact with older established workers. A single cohort of newly emerged worker (NEWs) containing > 3000 bees were marked with a paint dot on their thorax, and divided among healthy full-sized colonies containing thousands of older established workers, or nuc-box cages containing no older workers, different diet treatments and two 19X9.125 inch frames of drawn wax comb that had been previously exposed to older established workers. To control for the colony environment including exposure to older established workers, we returned 100 marked NEWs to each of three healthy growing colonies. We placed 300 marked NEWs into each of nine nuc-box cages (three treatments with three replicates), and provided the following diet treatments: freshly collected and stored pollen (beebread), autoclaved corbicular pollen, and no pollen (sucrose syrup only). Thus, exposure to older established workers only occurred for the colony control. The beebread diet treatment consisted of one frame with abundant freshly collected pollen obtained near the center of the brood nest from healthy colonies that were actively collecting and storing pollen. In choice tests, workers prefer 1–2-day-old pollen [49], so we provided fresh pollen that was likely collected that day or the previous day, as indicated by the lack of a honey cap, as is typical of older beebread. Both the colony control and fresh beebread diets are referred to as natural diets.

The autoclaved pollen treatment was comprised of corbicular pollen pellets stripped from the hind legs of foragers using a pollen-trapping device attached to the front of active colonies. To the corbicular pollen, we added 10% sterile H_2_O (w/v) and then worked the mixture into a pollen paste. This mixture was autoclaved and packed into a frame of empty wax comb. The no pollen treatment received a frame of empty wax comb. All non-social cages were given a 2nd 19X9.125″ frame of empty drawn wax comb to encourage clustering, and we provided sterilized 70% sucrose syrup and sterile water ad libitum via 30 ml drip bottles, and maintained the cages at 35 °C and 50% relative humidity. Both the autoclaved pollen and no pollen (sucrose only) diets are referred to as stressed diets.

After nine days, twelve bees from each cage or colony replicate were dissected and processed as described in Fig. 1 [48]. To capture intra-individual variation, we pooled three midguts, pylorus/ileums, or rectums per sample (library) prior to molecular analysis. We sequenced four libraries per replicate.

### PCR and MiSeq

For N = 48 pylorus/ileum and N = 48 rectum samples, we amplified the V6–V8 variable region of the 16S rRNA gene using PCR primers 799F (acCMGGATTAGATACCCKG + barcode) and bac1193R (CRTCCMCACCTTCCTC). DNA was amplified using the HotStarTaq Plus Master Mix Kit (QIAGEN, USA) with the following thermocycler program: 94 °C for 3 min, followed by 28 cycles of 94 °C for 30 s, 53 °C for 40 s and 72 °C for 1 min, with a final elongation step at 72 °C for 5 min. PCR products were confirmed on a 2% agarose gel. PCR products were then used to prepare DNA libraries via the protocol for Illumina MiSeq DNA library preparation. Sequencing was performed at the University of Arizona Genetics Core on a MiSeq following the manufacturer’s guidelines.

### MiSeq Sequence Analysis

Sequences were processed using MOTHUR v.1.35.1 [50]. The make.contigs command was used to join forward and reverse reads. We then removed the last five base pairs of the amplicon using the SED command in UNIX. We removed sequences containing ambiguous bases using the screen.seqs command, and unique sequences were generated using the unique.seqs command. A count file containing group information was generated using the count.seqs command. Sequences were aligned to Silva SSU Ref database (v102 [51]) using the align.seqs command. Sequences not overlapping in the same region and columns not containing data were removed using the filter.seqs command. Sequences were pre-clustered using the pre.cluster command. Chimeras were removed using UCHIME [52], and any sequences that were not of known bacterial origin were removed using the remove.seqs command. All remaining sequences were classified using the classify.seqs command. All sequences with only one or two (single/doubletons) associated reads were removed using the AWK command in UNIX. A distance matrix was constructed for the aligned sequences using the dist.seqs command. Sequences were classified with the RDP Naive Bayesian Classifier [53] using a manually constructed training set containing sequences sourced from the greengenes 16S rRNA database (version gg_13_5_99 accessed May 2013 [54]), the RDP version 9 training set, and all full-length honeybee-associated gut microbiota listed in NCBI (accessed July 2013). Operational taxonomic units (OTUs) were generated using the cluster command. Representative sequences for each OTU were generated using the get.oturep command.

### Bacterial and Fungal Quantification

Total bacteria and fungi in the midgut, pylorus/ileum, and rectum were quantified using the BactQuant and FungiQuant qPCR primers [55, 56] on Bio-Rad CFX96 thermocyclers. To provide absolute quantification of 16S or 18S rDNA copy number and ensure inter-run comparability, in-run standard curves and no-template controls were included on each run. Plasmid standards for each assay were created using either a 16S or 18S gene clone (using Invitrogen pCR®2.1-TOPO™ cloning vector (#K4500-40) and DH5α™ cells (#18,265,017) per manufacturer’s specifications), purified via plasmid mini-prep kit (Thermo Scientific #K0503), dsDNA/µl was determined via Implen NanoPhotometer P300, and the known mass of plasmid plus PCR insert was used to calculate 16S or 18S plasmid standard copies per µl. Standard curves were calculated from a tenfold serial dilution of the plasmid standards included on each run.

### Statistical Analysis

We calculated microbiota diversity by treatment and niche, using unique sequences identified in the bioinformatics pipeline. Using the summary.single command in Mothur, we rarefied to the smallest library (19,427 for ileum’s and rectum’s and 54,288 for the hindgut as a whole). We calculated observed number of unique sequences, Shannon’s (H), the Effective Number of Species or richness (ENS- defined as the exp(H)) and equitability or evenness of the microbiota defined as H/Hmax, where Hmax = ln(total # of OTU’s).

We used the top 196 OTUs accounting for > 99% of the reads to examine transmission. We first defined the top 196 unique OTUs by species, core hindgut membership and proportional representation by treatment. We then performed a test for proportions on the top 196 unique OTUs comparing the colony control to the treatments, and normal diets to stressed diets.

The microbiome data set was transformed by bactiquant results and species-specific rRNA copy number. To incorporate community size in the analysis, we multiplied the proportional abundance of OTUs returned by amplicon sequencing by the total bacterial 16S rRNA gene copies determined with qPCR for each individual tissue type. All core bacterial genomes contain four 16S rRNA gene copies except *Lactobacillus kunkeei* (5), *Bifidobacterium asteroides* (2) and *Bombella apis* (1). OTUs representing non-core diversity were summed and corrected for community size via mean (4.2) 16S rRNA gene copy number [57]. To allow the use of parametric multivariate analyses [58], we converted bacterial relative abundance to ratios among all OTUs [59] using the software CoDaPack’s centered log-ratio (CLR) transformation [60]. The analysis of centered log ratios is a measure of ratio abundance of a single taxon relative to the rest of the microbiota, so the data analysis reflects changes in the structure of the microbiota with respect to a single taxon. Principle component analysis (PCA) bi-plots were generated to explore the variance in the data. We performed a MANOVA comparing the hindgut (ileum and rectum) microbiota by treatment, corrected for multiple comparisons. We examined the CLR transformed data using two separate MANOVA models. The first analyzed the data as 4 separate treatments with colony or box nested in treatment. The second analyzed as the data as two diet treatments; normal diet (colony and pollen consumed the same diet) and perturbed diet, a group defined by significantly decreased consumption of fats, proteins and complex carbohydrates.

As a more straightforward measure of change in particular taxa without respect to other community members, we compare estimated cell counts by niche and taxon using an ANOVA performed on log-transformed cell counts normalized for species-specific 16S rRNA gene copy number. Effects attributed to diet were examined by comparing stressed diets (autoclaved corbicular pollen or no pollen) to normal diets of freshly collected beebread (colony and pollen). Microbial community structure was also compared with diet source and social context as factors. We compared bacterial and fungal copy number by niche using one-way ANOVA (Tukey HSD post hoc) and two-sample t-tests. We performed correlations examining log-transformed fungal abundance with each major bacterial taxon. All analyses were conducted in either JMP_v11(JMP_ 1989–2007) and/or SAS_ v9.4 (SAS Institute Inc., Cary, NC).

## Results

### Microbial Community Analysis

Next-generation sequencing returned 4,236,606 quality trimmed reads (400 bp) for the 96 libraries, an average of 44,131 sequences per library (Table S1). To distinguish *F. perrara* from *G. apicola*, we clustered the sequences at 99% similarity, producing a total of 4367 OTUs following the exclusion of singletons and doubletons. We then confirmed taxonomy via NCBI BLAST and consolidated core gut phylotypes from the top 40 OTUs reducing the data set to 8 core gut phylotypes. Following consolidation and taxonomic confirmation, eight of the top 40 unique OTUs (16, 22, 26, 30, 32, 33, 35, and 36) were sparse containing a high frequency of zero values. These eight OTUs were consolidated into a 9th group consisting of “other” (Σ OTUs 41–97 plus the eight listed above). Following consolidation, the eight phylotypes and “other” represented 97% and 1.5% of the total sequences, respectively, and were used for downstream statistical analyses. The dependent variable “other” is a combination of OTUs representing a measure of non-core bacterial abundance for each tissue.

### Microbiota and Mycobiota Size

Considering the three treatments where diet consumption could be measured, gut weight differed significantly by diet treatment [48], and was correlated positively with microbial community size across treatments (Rsq = 0.29, *p* = 0.0007). In the no pollen treatment provided only sucrose solution, the guts of nine-day-old adults weighed significantly less than the treatments provided pollen in some form, and newly emerged adults consumed 36% less autoclaved pollen than they did freshly collected pollen [48].

**Fig. 2:**
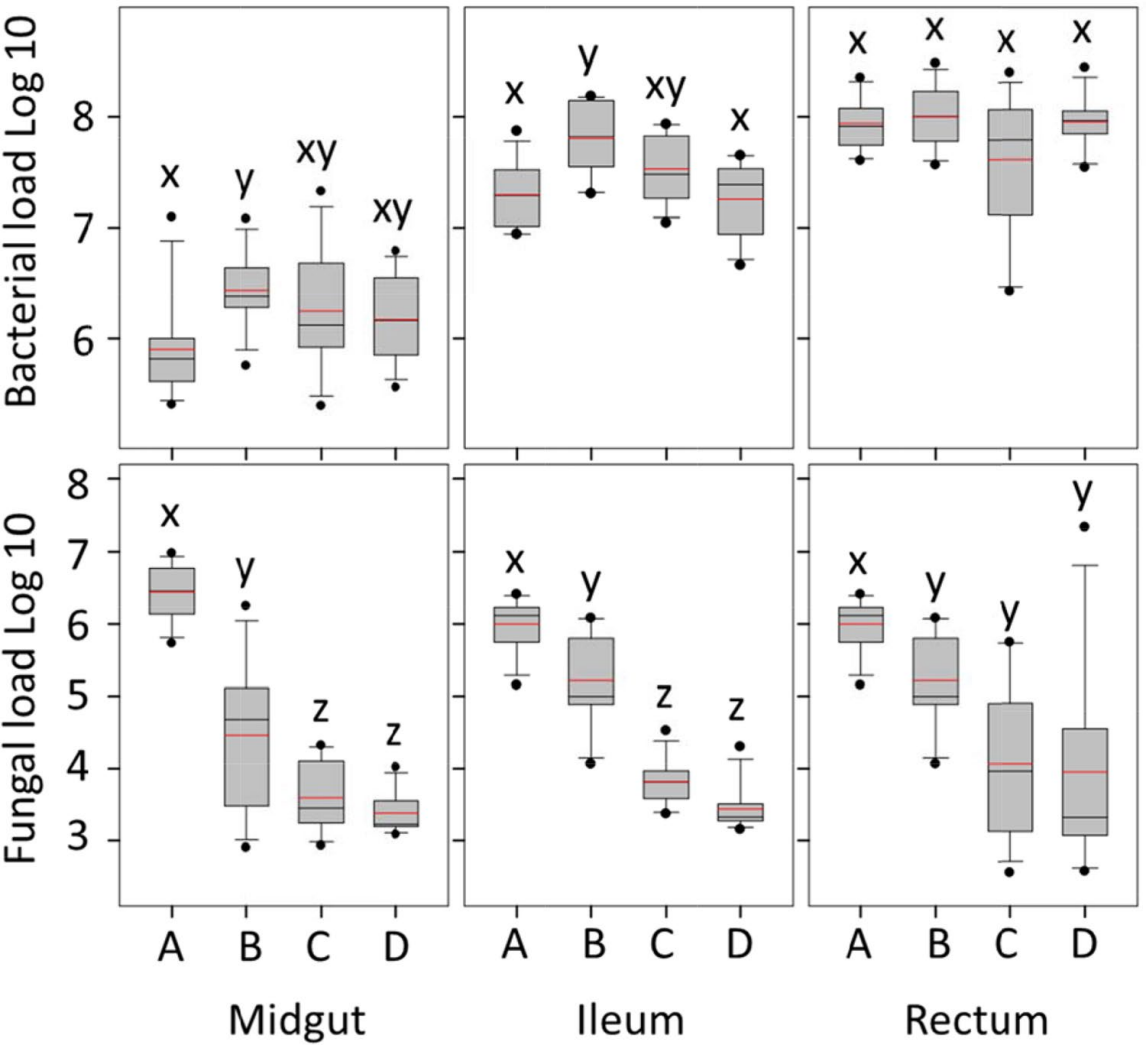
Bacterial and fungal load differ by social context and diet throughout the gut. On the x-axis, A is the positive control; large colonies exhibiting a typical age structure, and exposed to the pollination environment. Treatment B is fresh stored pollen (beebread), C is autoclaved corbicular pollen, and D is no pollen (negative control). Treatments B, C, and D lack the colony context, and each consist of three replicate cages containing 300 newly emerged bees and wax combs containing each diet treatment (Fig. 1). Y-axis values were determined by bactquant and fungiquant and are cell number for bacteria, rRNA copy number for fungi. Within each panel, distributions (boxplots) with the same lower case letter (x, y or z) do not differ significantly based on a Wilcoxon test (*p* < 0.05)

Bacterial abundance differed by treatment in two of three gut tissues (Fig. 2). Relative to the colony control, bacterial load was significantly greater in the midgut (F_3,44_ = 3.1, *p* = 0.04), and ileum (F_3,44_ = 7.9, *p* = 0.0002) of workers fed a diet of fresh beebread but raised in social isolation. In the midgut, bacterial abundance differed between the colony control and fresh pollen treatment (post hoc test, *p* = 0.02). In the ileum, the colony control differed in bacterial abundance from fresh pollen (*p* = 0.001), and no pollen (*p* = 0.0005). Bacterial abundance in the rectum did not differ by diet treatment or social exposure.

**Fig. 3:**
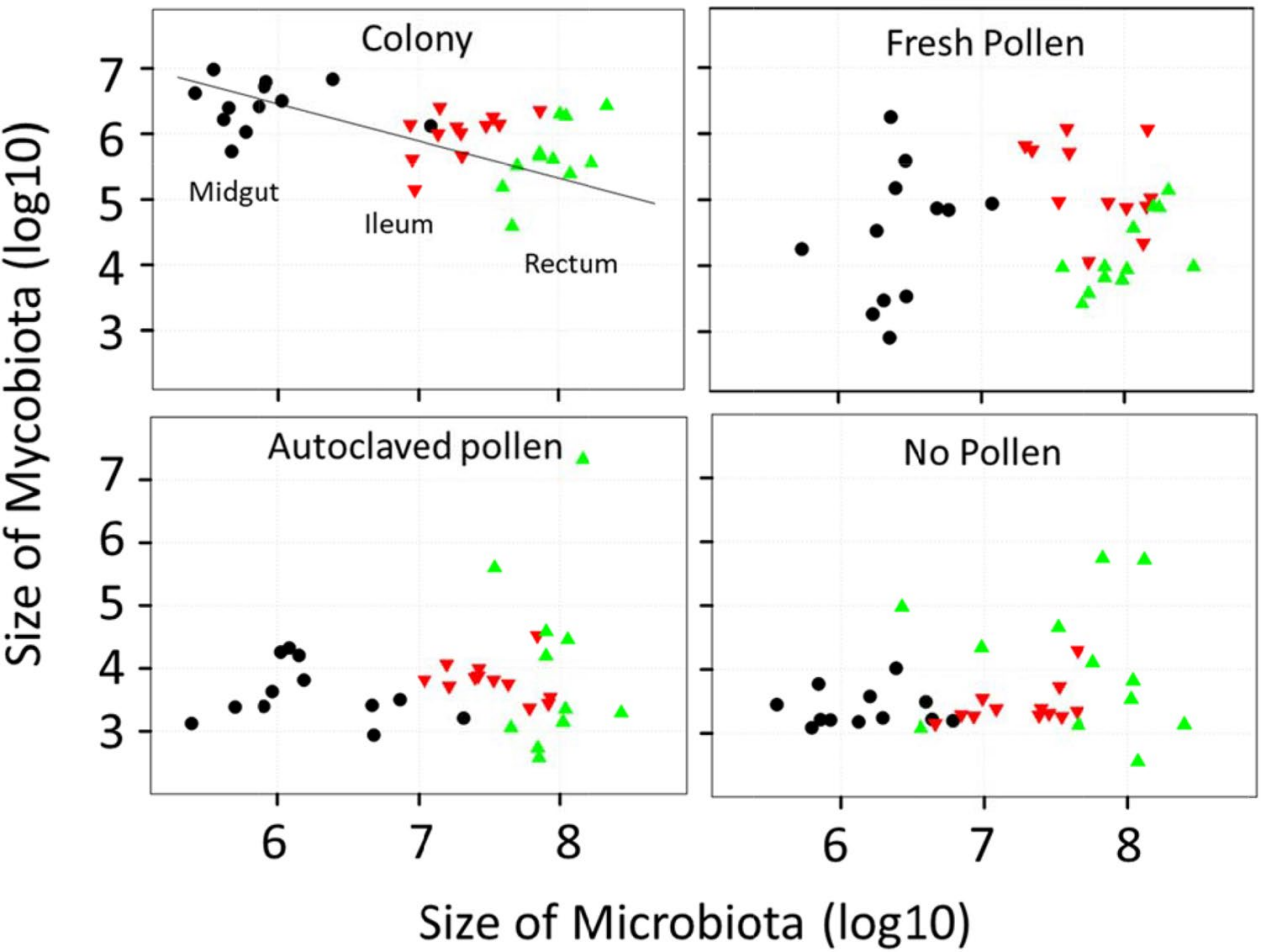
Throughout the alimentary tract, the presence of fungi (the mycobiome) or fungal associated factors was correlated with the structure of the bacterial microbiome. In our positive control, colony reared bees, there is a positive association of microbiota and mycobiota size, but considering the alimentary tract as a unit, fungal load decreases concurrent with increasing bacterial load (Rsq = 0.25, F = 11.4503, *p* = 0.002). This pattern shifted significantly when workers were detached from the active hive environment and provided various diet treatments. Values are from Fig. 2

Fungal abundance differed by treatment across all three gut sections (Fig. 2). In natural colony “control” bees, mycobiome size decreased significantly from midgut to ileum to rectum, and was significantly negatively associated with microbiota size considering the gut as a whole (Fig. 3). Relative to the colony control, fungal load decreased significantly in the midgut, ileum and rectum of bees fed fresh pollen. In the ileum and rectum, this difference was more pronounced for bees fed autoclaved pollen or no pollen (Fig. 3). Fungal abundance differed most drastically in the midgut (F_3,44_ = 64.3, *p* < 0.0001) as compared to the other two gut regions; post hoc tests revealed that colony control midguts differ from those of fresh pollen, autoclaved pollen and no pollen (*p* < 0.0001), and the fresh pollen treatment also differed from autoclaved pollen (*p* < 0.005) and no pollen (*p* < 0.0004). Fungal abundance also differed among treatments in the ileum (F_3,44_ = 90.8, *p* < 0.0001); post hoc tests show colony control differs from fresh pollen (*p* < 0.0004), autoclaved pollen and no pollen (*p* < 0.0001), and fresh pollen differs from autoclaved pollen and no pollen (*p* < 0.0001). In the rectum, differences were significant but less pronounced (F_3,44_ = 8.6, *p* = 0.0001). Following post hoc tests, fungal abundance in rectums of the colony control differed from fresh pollen (*p* < 0.002) autoclaved pollen (*p* < 0.0004) and no pollen (*p* < 0.0009).

### Microbiota Diversity

**Fig. 4:**
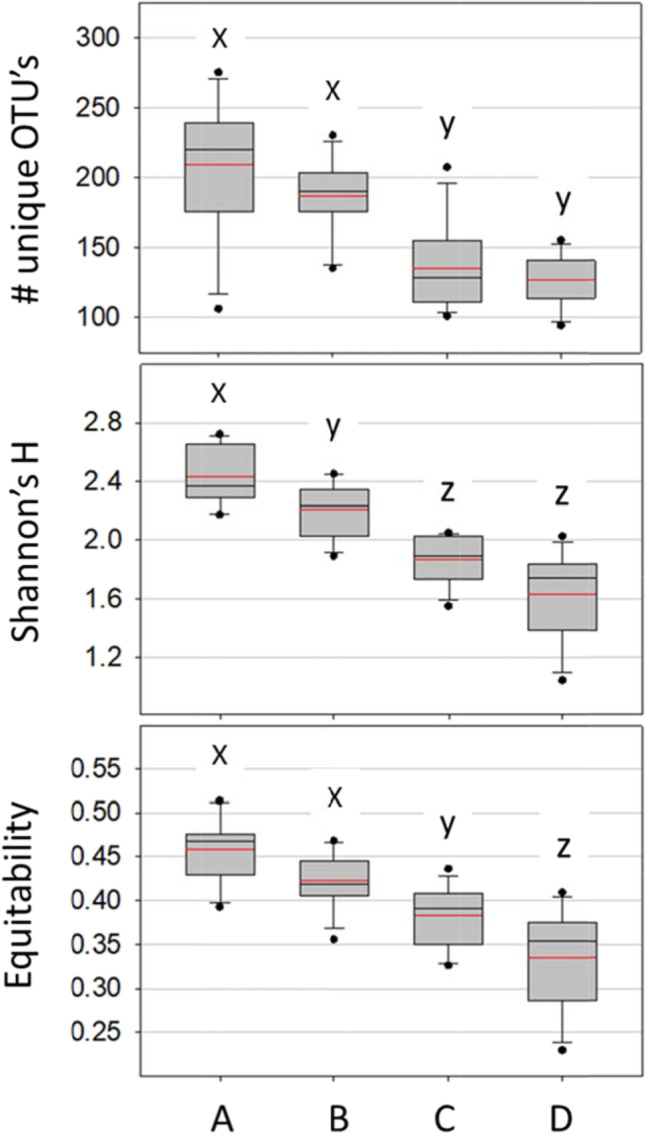
Diversity of combined hindgut tissues (ileum/pylorus and rectum) based on unique bacterial OTUs (top panel), Shannon’s H, and Equitability, a measure of evenness. The treatments on the x-axis are colony control (**A**), fresh pollen (**B**), autoclaved pollen (**C**), and no pollen (**D**). Within each panel, distributions (boxplots) with the same lower case letter (x, y or z) do not differ significantly based on a t-test (*p* < 0.05)

Based on unique bacterial OTUs, the diversity of combined hindgut tissues (ileum and rectum) differed by treatment including observed species, Shannon’s H, and Equitability, a measure of evenness (Fig. 4). Considering the detection of all unique OTUs, the colony control showed the greatest number of species, effective species, and evenness, followed by fresh pollen, autoclaved pollen and no pollen in that order.

Considering the top 196 unique OTUs, herein referred to as “establishment”, only the richness of *Gilliamella apicola* differed by social environment (Table 1). Hindguts from the fresh pollen treatment contained significantly fewer established OTUs than did the colony control: (18 vs. 47; one-tailed test of proportions Z = 5.6 *p* < 0.00001). The richness of the other four core hindgut groups did not differ by social environment, but *S. alvi* richness differed by diet treatment showing 21–22 established strains in normal diets, but only 11–13 in stressed diets (Table 1).

**Table 1:** Number of unique core hindgut OTUs established^a^ in the hindgut by treatment

Core hindgut bacteria	*Lactobacillus* Firm5	*Lactobacillus* Firm4	*Bifidobacterium* *asteroides*	*Snodgrassella* *alvi*	*Gilliamella apicola*	*Frishella perrara*	*Bombella apis*	*Lactobacillus kunkeei*	*Bartonella* *apis*	Other
**Total**	**41**	**6**	**20**	**27**	**55**	**4**	**18**	**12**	**2**	**11**
Colony Control	23	3	11	21	47	4	9	7	1	7
Fresh Pollen	28	4	16	22	18*	4	10	7	1	4
Autoclaved pollen	29	5	12	11^#^	10*	3	13	10	1	1*
No pollen	22	4	8	13^#^	18*	3	13	5	1	5

^a^ Establishment is roughly defined as the 196 most abundant unique OTUs in the combined hindgut tissues

^*^Treatment significantly different from the colony control based on a test for proportions (*p* < 0.00001)

^#^ Treatment significantly different from fresh pollen and colony control based on a test for proportions (*p* < 0.01)

### OTU Differences by Treatment

#### Colony Control

**Fig. 5:**
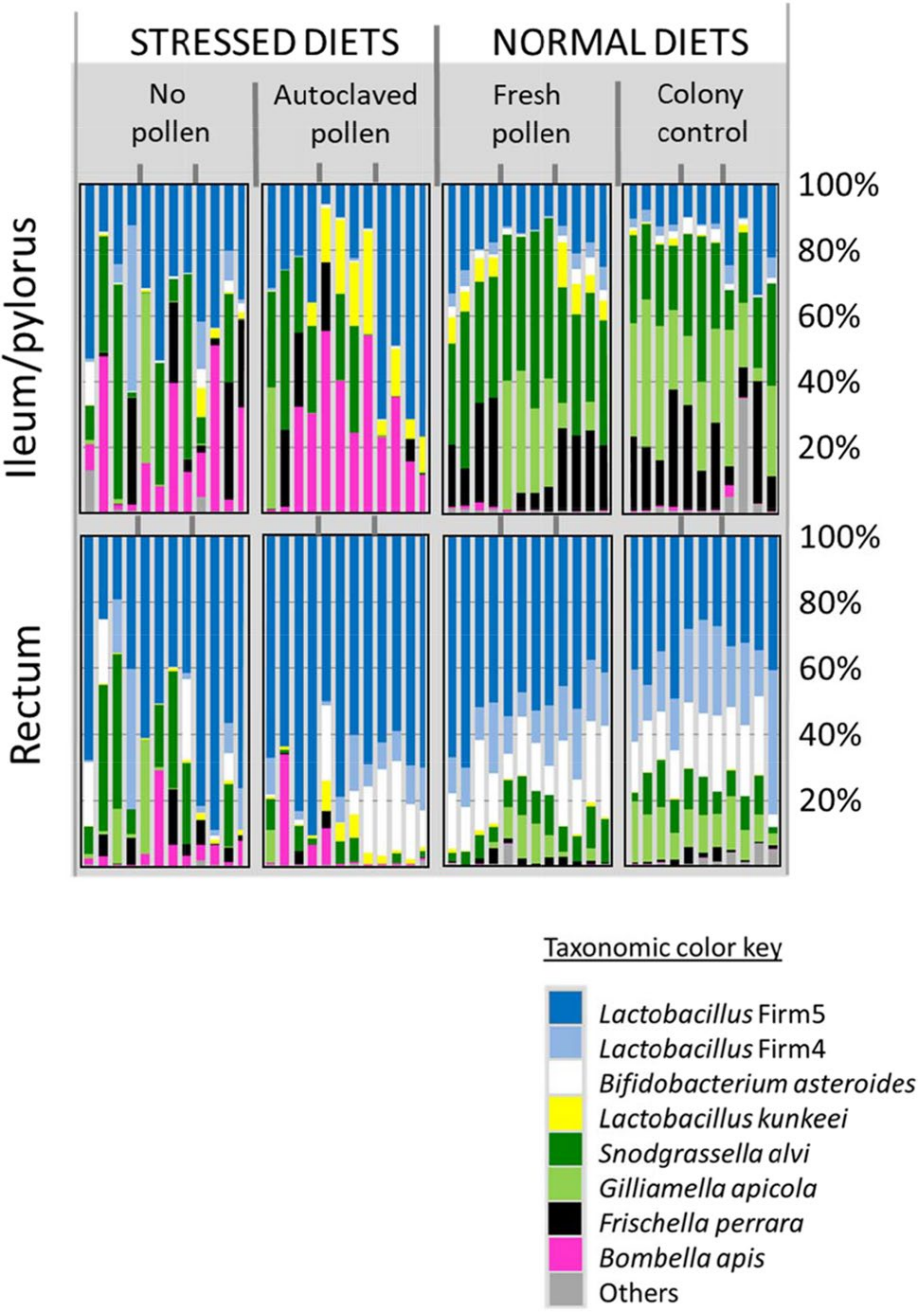
Bar charts representing the relative abundance of bacterial OTUs from two hindgut tissues and three treatment conditions plus a control, each composed of three replicate cages or colonies marked by grey dividers at the top and center of the diagram. Each bar is a single Illumina library, representing the combined tissues of three workers. We sequenced four libraries per replicate. The colony control is a natural colony environment. Diet treatments lack the colony context. The vertically oriented ileum and rectum microbiotas correspond to the same worker individuals. Note replicate variation

NEWs that we returned to the colony environment less than three hours after emergence developed a typical gut microbiota characterized by evenness of the core microbiota in both the ileum and rectum (Figs. 4 and 5). All of the core species were present in relative and absolute abundance typical of a nurse worker sampled from the natural hive environment. The microbiota was characterized by high equitability among core species including core ileum species *F. perrara* and *G. apicola*. Fungi were strongly associated with microbiota structure and taxonomy throughout the gut of the colony control (Fig. 3). The mycobiota and microbiota showed strong associations throughout the alimentary tract, with fungi decreasing in absolute abundance moving towards the rectum concurrent with increasing microbiota density. The occurrence and evenness of *Frischella perrara* was associated with “bioactive” pollen consumption in both the colony control and fresh-stored pollen treatment (Fig. 5).

#### Fresh-Stored Pollen

**Fig. 6:**
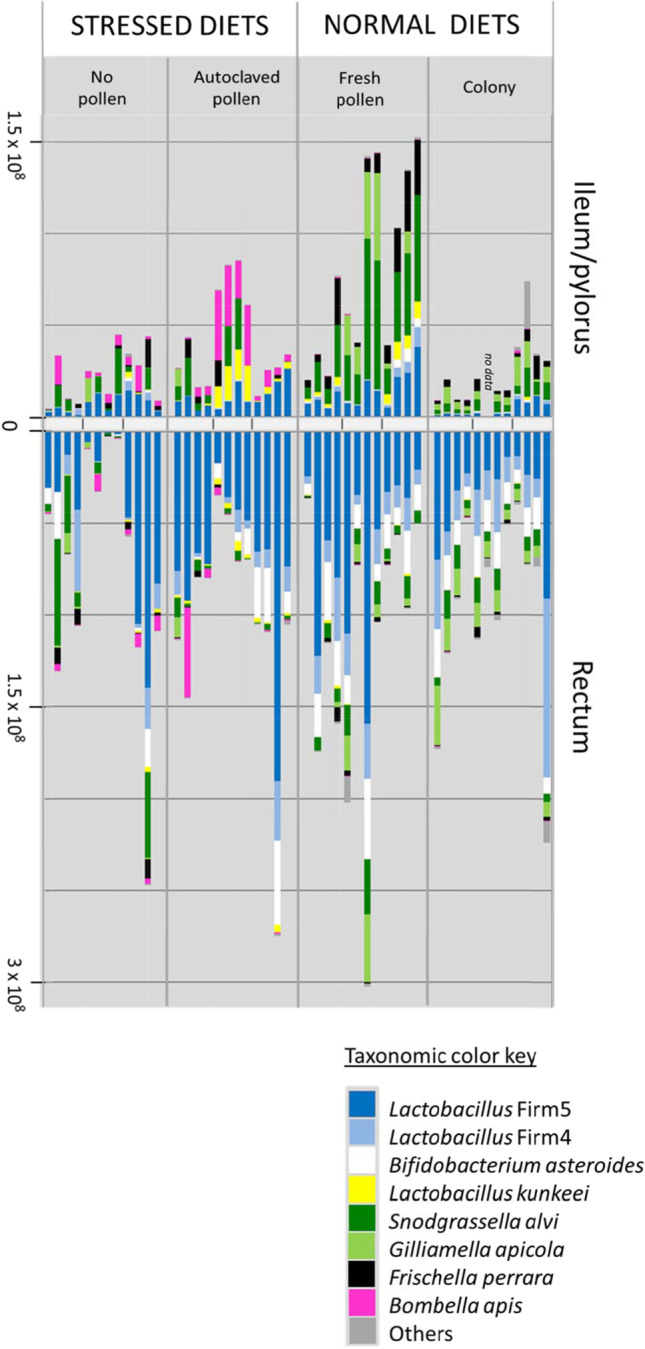
Hindgut Microbiota of 9-Day-old workers reared in large functional colonies or in social isolation with different diet treatments and a control. Bar charts represent the Bactquant normalized abundance of bacterial OTUs from two hindgut tissues (ileum/pylorus and rectum). Colony is the positive control; a natural outdoor colony environment. The three diet treatments lack the colony context. Each bar is a single Illumina library, representing the combined tissues of three workers. We sequenced four libraries per cage or colony replicate. The x-axis is centered at zero and increases in absolute value moving in either direction

Relative to the colony control, newly emerged bees removed from social/colony contact but provided a diet of fresh-stored pollen showed significantly larger bacterial communities in the midgut and ileum, and changes to both absolute and relative abundance of core OTUs (Fig. 6). Found primarily in the ileum, and important for gut function, *G apicola* was abundant in only one of three fresh pollen replicates. However, with the exception of *G. apicola*, the taxonomy and relative abundance of all other core hindgut species resembled that of the colony control (Fig. 5). In the ileum, *L. firm5* increased in both relative and absolute abundance, while *G. apicola* and total fungi decreased (Fig. 6, Table S2)*.* The rectum microbiota of the fresh pollen treatment was almost indistinguishable from that of the colony control, but fungi was significantly reduced, and the fungal-bacterial association was disrupted throughout the alimentary tract relative to the colony control (Figs. 2 and 3).

#### Autoclaved Pollen

We designed this treatment to provide pollen nutrients, but exclude any biotic activity from the pollen itself, or microbes in the corbicular pollen pellet. Thus, it is a control of sorts for the fresh pollen diet. However, the consumption of autoclaved pollen was significantly less (63%) than that of fresh-stored pollen [48], and subsequently, microbiota size in the ileum was significantly associated with diet consumption, measured as gut weight. Even so, with the consumption of solid food, the microbiota still established in a tissue-specific manner, with the ileum composition differing from that of the rectum (Fig. 5). Relative to the colony control, this treatment resulted in similar sized microbiotas, but significantly smaller mycobiotas throughout the gut (Fig. 2). Both absolute and relative abundance of core OTUs differed from the control including significant increases in the ileum of *L. firm5, L. kunkeei, and Bombella apis*, and decreases of *G apicola*, *S. alvi* and *F. perrara* (Figs. 5 and 6, Table S2). *G apicola* was virtually absent from this treatment establishing in only a single individual. When compared to the fresh-beebread diet, differences were similarly pronounced for both hindgut tissues (Table S2).

#### No Pollen

With brief exposure to the colony environment but no pollen nutrition, size of the midgut, ileum and rectum microbiotas did not differ from the colony control (Fig. 2). This treatment differed taxonomically from the control and other treatments for many core OTUs, including *L. firm5, L. kunkeei, G. apicola and Bombella apis*, in the ileum and *L. firm 4* and *B. asteroides* in the rectum (Figs. 5 and 6, Table S2). In general, the taxonomy and structure of the ileum microbiota was similar to that of the rectum, and the diversity indices indicate less diverse, dominance communities relative to the colony control and other treatments (Fig. 4).

## Discussion

Our results build on previous findings [8, 9, 35] and suggest that both diet and colony exposure are critical for generational transmission and microbiota assembly (Figs. 2 and 3). Workers excluded from natural eclosion and frame exposure and then fed sterile pollen in sterile environments established hindgut microbiotas too small for next generation sequencing, suggesting little vertical transmission and poor establishment [8]. Here, we demonstrate that natural eclosion, brief exposure to the emergence frame, and a diet of fresh beebread resulted in the assembly of adult hindgut microbiomes highly similar to those that established in the colony environment (Figs. 5 and 6). The primary difference when excluding the colony environment was a sharp decline in fungal abundance, and a 50% failure of *Gilliamella apicola* to establish in the ileum. Establishment *G. apicola* may require extended exposure to the colony or hive environment so the host and incipient microbiota can “sample” multiple strains of *G. apicola* compatible with themselves, and the pioneer gut residents that establish prior to *G. apicola*; *L. firm5* and *S. alvi* [8, 9].

Below we argue that core hindgut microbiome transmission in honey bees is not reliant on social transmission, but is best defined as facultative horizontal because it occurs readily in the absence of older workers, and is acquired largely via diet and emergence frame exposure (Fig. 5). In general, our hypothesized transmission mode resembles that of the marine environment [61], because it appears difficult for NEWs to avoid horizontal transmission from the hive environment. The near obligate mode in marine environments, horizontal transmission is considered beneficial for the maintenance of host–symbiont interactions because it creates competition among symbionts and allows partner choice, including mechanisms of host reward and punishment [62]. Based on mathematical models, partner choice is predicted to require horizontal transmission so hosts (and perhaps early symbionts) can choose between subsequent symbionts [63]. Because no single experiment is definitive, we discuss our results broadly with careful attention to experimental context and design applied in separate studies. Given the nature of our results, we discuss the potential associations of fungi with microbiota assembly, and two major distinctions suggested by the dependent variables; a comparison of social to asocial environments, and natural to stressed diets.

### Transmission and Assembly

The two major experiments testing vertical transmission between honey bee worker generations concluded that social interaction of NEWs with older workers either had little effect, or did not produce a typical hindgut microbiome [8, 9]*.* Our present results agree and suggest that NEWs acquire their core hindgut bacteria from natural eclosion, frame exposure and the consumption of fresh beebread [17, 20]. Foragers likely inoculate fresh beebread with the core hindgut bacteria while packing pollen into corbiculae and while unloading pollen into the hive storage cell. Foragers have defecated at least once, but the frequency during pollen collection is unknown. The simple act of pollen collection may move continual trace amounts of pollen through the gut [64] where they are inoculated with core bacteria. Foragers gather pollen by cleaning it off their body hairs with fine-toothed leg combs often contacting the distal abdomen. This process can require hundreds of repetitions to load a single forager with gathered pollen. Honey bee workers do not practice proctodeal (mouth to cloaca) trophallaxis [26], but during flight, freshly collected pollen is first mixed with honey and hypopharyngeal gland secretions before it is packed into the corbiculae using the mouthparts and a series of leg to leg transfers. The entire suite of core gut bacteria can be found on the mouthparts, and in the hypopharyngeal gland of older bees [21, 23].

Returning newly emerged bees to the active colony environment provides contact with various hive substances related to local and hive-specific biotic conditions, microorganisms, secretions and behaviors, known disease and opportunists. Because newly emerged worker bees perform emergence behaviors as part of their life history, all treatments were allowed natural eclosion and brief (< 3 h) exposure to the emergence frame. Given this initial exposure, the asocial fresh pollen treatment was highly similar to the social colony control based on multiple measures (Figs. 2, 3, 4, 5 and 6). In the stressed and sterilized diet treatments, we detected significantly less fungal abundance and bacterial diversity (Figs. 2 and 4). Under normal conditions, newly emerged adult worker bees chew out of their pupal casings, clean cells from the larval nest area and then consume pollen or available nutrition [9, 26]. Following this exposure, both diet and social isolation significantly affected the establishment of hindgut microbiota explaining similar amounts of variation (Figs. 5 and 6, Table S2). In agreement with past work [8, 44], the greatest diversity and evenness occurred in the colony control, but total richness and evenness of the fresh-pollen treatment did not differ significantly from the control colonies (Fig. 4). This suggests that the majority of the hindgut microbiota is readily acquired by NEWs sampling a small fraction of the available hive space then consuming freshly-collected pollen. While transmission can occur primarily via these activities, the factors that govern microbiome assembly apparently rely on extended exposure to the active colony environment. Early life behaviors are often critical for microbiome establishment and subsequent health in animals [65], and behavior of honey bee workers separated from the active colony environment is abnormal, confounding attempts to distinguish social vs. hive exposure.

We analyzed the top 196 OTUs as a metric of establishment and found that the fresh pollen treatment contained a greater variety of core hindgut OTUs than did bees from the control colony with the single exception of *G. apicola* (Table 1). This suggests that transmission was similar, but the process of assembly differed. When bees consumed fresh beebread in social isolation, five of six core hindgut species had similar patterns of prevalence, occurrence and diversity in the hindgut. Only the establishment of *G. apicola* seemed to rely on extended exposure to the active colony. Moreover, we observed that only ileums containing two to four unique *G. apicola* sequences seemed to establish at all regardless of treatment, suggesting that *G. apicola* establishes best as an interspecies partnership or that various *G. apicola* strains are partitioned by niche or resource availability. With a diet of fresh pollen in social isolation, *S. alvi* bloomed in the pylorus/ileum increasing by nearly an order of magnitude relative to the colony control. *S. alvi* growth is suppressed by *G. apicola* in vitro, [66]*,* perhaps explaining in part the increase in *S. alvi* abundance associated with limited exposure to the active hive environment (Fig. 6).

*S. alvi* is functionally associated with *G. apicola*, and the abundance of these two species correlate strongly and predictably in the ileum of normal colony-reared bees [67] (Fig. 5). *S. alvi* is associated with the gut lining of the ileum, where it is hypothesized to consume and mitigate host supplied oxygen [4, 38]. Under normal conditions, or when colonized by a “conventional” microbiota, the central region of the ileum becomes anoxic whereas oxygen remains detectable in the center and periphery of “germ free” bee ileums fed sterile pollen [4]. Data on foragers suggest this relationship may be variable or change with age or diet [68]. Strongly allied with the host epithelium, *S. alvi* contributes to biofilm life, including SCFA and siderophore production [38, 69]. Based on our analysis of the top 196 unique OTUs and other work, diversity of *G. apicola* strains is deep relative to its abundance in the hindgut system suggesting strong competition for partnership with *S. alvi* (Table 1). Confirming earlier hypotheses [67], the significant abundance of established *G. apicola* variants relative to the other core members suggests that the early ileum ecosystem and/or *S. alvi* specifically selects for *G. apicola* diversity of function (Table 1). Correspondingly, *G. apicola* strains show notable variation across many functional categories including oxidative stress and carbohydrate utilization, while *S. alvi* strains are typically fixed for similar functions indicating competition and host fidelity respectively [38, 48, 70–72].

Confirmed in separate laboratory experiments, both *S. alvi* and *G. apicola* establish poorly when NEWs were exposed to either five or 300 older siblings and wax frames of unstated origin [8, 9]. Even when directly feeding isolated NEWs with fresh macerated adult hindgut contents mixed with pollen, *G. apicola* established poorly relative to the colony control, attaining typical proportions in < 50% of individuals [8], mirroring our results for fresh-stored pollen (Figs. 5 and 6). Collectively these results suggest that *G. apicola* establishment is sensitive to a socially related factor. Positively correlated with the abundance of *G. apicola,* fungal density was significantly diminished throughout the gut, suggesting that fungi are also difficult to acquire with limited exposure to the colony environment. More control of microbial exposure is required to test this hypothesis. If this particular bacterial partnership does not establish, it appears to trigger a cascade of negative changes in host physiology [21, 44]. Exposure to a greater variety of *G. apicola* strains will increase the chance of compatibility with sister strains, and ileum partner *S. alvi*.

**Fig. 7:**
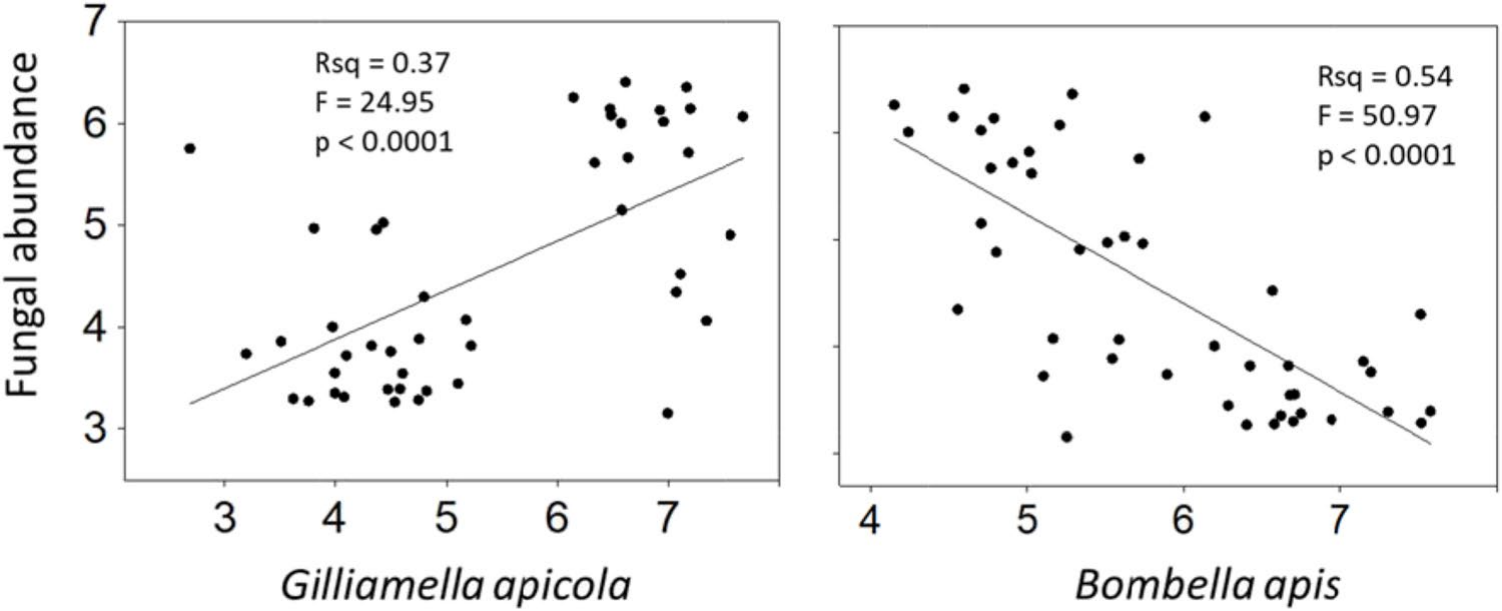
The significant relationship of fungal abundance with two different bacteria in the worker ileum. Trend line represents Pearson’s correlations of log transformed abundance measures. Axes are displayed as (log 10) exponents. Fungi are 18S rRNA copy number, and bacteria represent cell counts, normalized by genome-specific copy number

*G. apicola* and/or the ileum partnership with *S. alvi* may be functionally “replaced” by *L. kunkeei* and *Bombella apis* under various environmental conditions (Fig. 5), and more generally across studies of microbiota variation examining diet, stress and social exposure [32]. *L. kunkeei* and *Bombella apis* populate the queen ileum and tend to co-occur with a positive association across many honey bee niches [3, 23, 73]. Although their abundance in worker guts is associated with host deficiencies, these species may buffer highly pathogenic microbial changes in the gut. *Bombella apis* and *L. kunkeei* are sugar fermenting aerobes or micro-aerobes, negatively associated with fungal density in the rectum and ileum respectively (Table S2, Fig. 7). Their abundance in the hindgut may indicate oxygen availability, a dysbiotic condition. Based on the results of a companion study examining enzyme properties of these same samples [48], the dysbiotic workers consuming stressed diets suffered significant depletions of head gland proteins, a critical marker of development in 9-day-old worker bees. This represents a significant cost to the colony, because NEWs cannot perform the duties of a young nurse, and will likely contribute little resources as an older forager. Perhaps most informative, this entire nutritional microbiota dynamic was strongly related to fungal abundance, a factor long ignored in microbiome studies of the honey bee gut and other organisms (Figs. 1 and 2).

### Fungal Relationships

The variation in our results attributable to diet (Fig. 2) indicates that a fungal inoculum is obtained from exposure to the active colony environment and hive-stored pollen (Figs. 2 and 3). The significant depletion of gut fungi in the stressed diet treatments was associated with a variety of revealing microbiome changes (Fig. 4). Although not explicitly tested, we are confident that bacteria and fungi present the stressed diets were killed by autoclaving, consistent with our results for microbiota density and diversity (Figs. 2 and 4). *Bombella apis* was abundant and diverse in the dysbiotic ileum microbiomes resulting from sterilized and stressful diet conditions (Figs. 5 and 6). Across the data set, *Bombella apis* became abundant when fungi were sparse suggesting competitive release. In support of this, *Bombella apis* is a fungal antagonist demonstrated to inhibit the growth of *Aspergillus flavus*, a mycotoxin producing mold ubiquitous in beebread [74]. Similarly, *Bombella apis* abundance across the data set was negatively associated with all three core ileum species *F. perrara, G. apicola* and *S. alvi* (Table S2). Notably, these are the bacteria hypothesized to require social transmission by earlier investigations [8, 9]. For comparison, the ileum of the honey bee queen is a low fungus environment that supports *Bombella apis*, *L. kunkeei* and *Lactobacillus* firm5, and occasionally *S. alvi,* but not *G. apicola,* or *F. perrara* [3]. Both *L. kunkeei* and *Bombella apis* were significantly more abundant in the stressed diet treatments. Diet is the difference between worker and queen phenotypes [28], suggesting microbiome and mycobiome evolution in the gut were sculpted in part by this factor. Collectively, patterns in the dependent variables combined with known bacterial and gut function suggest that fungi or fungal associated factors contribute to core-hindgut microbiota assembly especially in the ileum (Fig. 3). Our data are also consistent with resident yeasts in the worker gut [47] inhibiting the establishment of *Bombella apis* under natural diet conditions (Fig. 7), although this remains to be tested.

Fungi and bacteria are considered competitors for simple plant-derived substrates, and antagonistic and mutualistic interactions have evolved for recalcitrant substrates found in pollen such as cellulose and lignin. Aerobic cellulose degradation is ubiquitous among fungi whereas anaerobic degradation of cellulose is common among bacteria that inhabit the anoxic digestive tracts of animals [75]. A vast variety of fungi occur in hive-stored pollen and floral nectar including osmotolerant yeasts that briefly bloom in 1–2 day-old fresh pollen [76]. Although fungi were rare in our samples, they were strongly associated with keystone ileum species, *G. apicola* (Fig. 7), a symbiont with functional diversity for recalcitrant pollen substrates [77]. *Saccharomycetes* are known to colonize the nurse bee gut [46], and some appear to be vertically transmitted, surviving pupation to proliferate in adults [47]. Recent work on the first pass infant meconium in humans suggests that the mycobiota also begins at birth, and despite their rarity, fungi are involved in critical host functions and opportunistic disease [78].

The collection of recent results supports the existence of a rare fungal community that populates the gut of honey bee workers [46, 47, 68]. In agreement with another recent study [68] we found a strong relationship between bacterial and fungal density throughout the alimentary tract in the colony control, perhaps a reflection of broad changes in gut pH and oxygen (Fig. 3). As a bioindicator of oxygen availability in the colony control, fungal density decreased and bacterial density increased approaching the rectum concurrent with the shift from aerobic to anaerobic metabolism [69]. Within each gut niche however, bacterial and fungal abundance were positively associated suggesting a lack of antagonism. This relationship was pronounced in the colony control, lost in the fresh pollen treatment, and reversed in the stressed diet conditions wherein fungi was most abundant in the rectum (Figs. 2 and 3). While these patterns suggest a shift in gut physiology [4, 68], more detailed experiments are required to test this hypothesis. Results from foragers indicate variable oxygen availability in the rectum [68], but core bacterial genome function suggests resilience to these fluctuations [79].

The rectum microbiota of NEWs fed normal diets was almost indistinguishable from that of the colony control, establishing similarly regardless of ileum changes (Fig. 6). The rectum communities associated with stressed diets featured atypical representation of core species with reduced abundance of both fungi and core bacteria *Lactobacillus* firm4 and *Bifidobacterium* (Figs. 2 and 6). Across the data set, Fungi associated positively with these OTUs suggesting synergy of function in the hindgut (Table S2). Almost exclusive to the rectum of healthy worker bees, *Bifidobacterium* is a facultative anaerobe equipped to exploit a large swath of recalcitrant polysaccharides considered prebiotic carbohydrates that support the maintenance of healthy gut flora [48, 77, 80]. Polysaccharide enzyme activity was significantly depleted in the hindguts of NEWs fed the stressed diet treatments [48], and *Bifidobacterium*, a ubiquitous fermentative constituent of healthy gut flora, was largely absent in half of the samples (Fig. 6).

How does fresh pollen consumption affect microbiota establishment? Relative to the fresh pollen fed treatments, the lack of solid food (sugar only) resulted in a sporadic pattern of species co-occurrence and increased microbiota similarity between in the ileum and rectum (Figs. 5 and 6), suggesting that the tissue specificity of hindgut colonization may depend in part on the consumption of high nutrient solids (pollen or pollen substitute). Consumption of the autoclaved pollen diet treatment produced gut communities intermediate between fresh pollen and sterile sucrose syrup. Despite hydrating and autoclaving, workers ate the autoclaved pollen diet at 63% of the fresh pollen diet [48]. The pollen (or solid) diet may affect peristalic motility, or the production of midgut peritrophic matrix, processes that mitigate microbiome establishment in other model insects [81]. Freshly stored pollen is enzymatically active, releasing carbohydrate-active enzymes involved in plant cell wall modification into the bee digestive tract upon consumption [48]. Similarly, recent work suggests that fresh pollen consumption is associated with the release of reactive oxygen species [64], conditions that may favor the establishment of the core hindgut bacteria. In addition to limiting microbial exposure, autoclaving bee collected pollen certainly altered the palatable properties of pollen including pH, solute concentration and pollen vitality. Similar to a previous study, natural diets were associated with uniform *F. perrara* establishment and niche specificity for the pylorus [36], associations completely lost in the stressed diet treatments (see also [8]). While *S. alvi, G. apicola* and *F. perrara* all populate the pylorus [36], fungal load had the strongest relationship with *F. perrara* abundance, again highlighting the potential contribution of fungal associated factors to microbiome assembly.

*Lactobacillus* firm5 is found with high prevalence and abundance throughout the hive environment and is readily acquired with natural eclosion [9, 17, 20, 82]. It also dominates the hindgut microbiota and is a functionally variable phylotype thought to include at least four gut prevalent species [83]. Based on detailed genome analysis from two locations, four functionally different species of the *Lactobacillus* firm5 phylotype are abundant in every hindgut, and hypothesized to coexist via resource partitioning [83]. Although less precise, our unique sequence analysis based on distinct gut regions supports the coexistence of 2–4 major strains of firm5 in the hindgut, but also suggests that *Lactobacillus* firm5 may associate by niche. While our 400 bp sequence cannot distinguish among three of the four major *Lactobacillus* firm5 species, OTU2 corresponds to *Lactobacillus apis* which appears to colonize first [9], and preferentially colonize the ileum under normal diet conditions. This suggests *L. apis* niche fidelity for the ileum, perhaps via adaptations to exploit host-excreted nutrients. *Lactobacillus apis* -host fidelity was disrupted in the stressed diet treatments but the same group of unique *Lactobacillus* firm5 sequences dominated the hindgut in a different way; stressed diet treatments tended towards greater evenness of *Lactobacillus* firm5 species, while microbiotas associated with normal diets tended towards greater dominance.

## Conclusion

Relative to vertical transmission, a mechanism of facilitated horizontal transmission may be a more robust evolutionary strategy because it promotes increased population variability, allows for partner choice, and results in greater competition within and between species/strains [62]. Many factors, including a high degree of strain variability [84], are consistent with transmission from various active hive refugia, followed by assembly that relies on partner choice. The competition for transmission and survival in the hive environment likely promotes an ever-changing pool of functional strain variants. If exposure to various competing strains leads to greater microbiome competence, then mechanisms like built structures that expand and diversify transmission space would be beneficial [61]. Facilitated horizontal transmission may occur in many host species that build extended structures as part of their life histories [7]. In honey bees, the hive itself might be considered the maternal microbiome, a transmission source filled with the complete hindgut microbiome signature. As hypothesized [8], the presence of active hive related factors may represent a form of social immunity, limiting microbiota size and modulating membership in the hindgut, particularly the ileum.

While many microbes are introduced from the pollination environment, the active hive environment does not represent an alien microbiome composed of non-specialized residents; it is comprised in large part of microbes carried with worker bees when they reproduce by budding [20]. These colony microbes then populate the built hive environment. When filled with stored food and developing young, the hive environment provides a variety of niche characteristics similar to those encountered in gut environments including pH, oxidative and osmotic stress, and various antimicrobial agents [16, 17, 85]. This built environment allows selection to operate simultaneously on an extended pool of population variants, yet retain a level of fidelity required for host-symbiont co-evolution. As an example, the two primary species of bacteria that dominate the hive environment, *L. kunkeei* and *Bombella apis,* are also core species that colonize the queen ileum [3, 40, 86]. Newly emerged queen bees do not contact their mother queen, so by deduction, the single most important member of the hive acquires their gut microbiota before/during adult emergence and/or via the hive/social environment.

Our results show that hindgut microbiota assembly involves both fungi and bacteria, and relies on factors associated with diet and active hive exposure. With few exceptions, fungi have been ignored in studies of the honey bee microbiota [46, 47]. Symbiotic relationships between Hymenoptera and fungi are numerous [87], and the relationship between bacteria and fungi in the gut is an emerging area of study [88]. Based on our findings, it seems quite likely that the much smaller fungal microbiota co-occurs with the bacterial biofilm, and interacts with the host and bacterial microbiota as in other exemplars [89].

## Supplementary Information

Below is the link to the electronic supplementary material.

Supplementary file1 (XLSX 215 KB)

Supplementary file2 (XLSX 930 KB)

## Data Availability

Next gene sequencing libraries of the ileum and rectum were deposited in GenBank under Sequence Read Archive (SRA) submission number SUB10690181.
